# Central Pancreatectomy as a Surgical Alternative for Parenchyma Preservation

**DOI:** 10.7759/cureus.75081

**Published:** 2024-12-04

**Authors:** Camila Sotomayor, Herta N Sagredo, Alessandra Jarufe, Eduardo Viñuela, Nicolás Jarufe, Jorge Martínez, Eduardo Briceño, Martín Dib

**Affiliations:** 1 Department of Hepatobiliary and Pancreatic Surgery, Pontificia Universidad Católica de Chile, Santiago, CHL; 2 Division of Transplantation, Department of Surgery, Beth Israel Deaconess Medical Center, Harvard Medical School, Boston, USA

**Keywords:** central pancreatectomy, hepato-pancreato-biliary surgery, open pancreatectomy, pancreas tumor, pancreatic neuroendocrine tumor (pnet)

## Abstract

Pancreatoduodenectomy and distal pancreatectomy are standard treatments for various pancreatic pathologies. These procedures involve radical resection and a significant loss of pancreatic tissue, which can lead to exocrine and/or endocrine pancreatic insufficiency.

In selected cases of benign tumors or those with low malignant potential, central pancreatectomy can be performed with acceptable morbidity and mortality rates. The advantage of preserving the maximum amount of healthy pancreatic tissue is the retention of both exocrine and endocrine pancreatic function.

We present the case of a 45-year-old female patient with a history of conversion from sleeve gastrectomy to Roux-en-Y gastric bypass three years prior due to gastroesophageal reflux disease (GERD). She presented with a pancreatic cystic lesion incidentally detected during abdominal ultrasound screening. Magnetic resonance imaging (MRI) revealed a 20 mm cystic lesion in the neck of the pancreas without signs of aggressiveness. Endoscopic ultrasound showed no features suggesting malignancy, with aspirated citrine-colored fluid, carcinoembryonic antigen (CEA) < 1.8 ng/mL, amylase of 144 U/L, glucose of 102 mg/dL, and cytology positive for neuroendocrine tumor of the pancreas (pNET). A PET scan with octreotide showed hyperuptake in the pancreas, with no evidence of additional lesions. An open central pancreatectomy was performed without complications. The patient had a favorable postoperative course and was discharged on day 5 without a pancreatic fistula. Biopsy confirmed a well-differentiated 2.1 cm grade 1 neuroendocrine tumor (G1 NET). Surgical margins were negative, with no vascular, lymphatic, or perineural invasion (pT2N0). At the five-month follow-up, the patient was asymptomatic, with a control abdominal PET-CT showing no abnormalities.

A retrospective review of the patient's medical records and a literature review were performed.

## Introduction

Central pancreatectomy has emerged as an effective therapeutic alternative for the management of benign and low-grade pancreatic tumors, especially in cases where the preservation of pancreatic function is crucial, and minimizing morbidity associated with more radical resections, such as distal pancreatectomy or pancreatoduodenectomy, is desired [1]. Unlike these traditional techniques, central pancreatectomy allows for the resection of localized neoplasms without significantly compromising the surrounding pancreatic tissue, resulting in a reduced risk of postoperative pancreatic insufficiency [2]. Recent studies support central pancreatectomy as a valid therapeutic option in selected clinical contexts, where multidisciplinary preoperative evaluation plays a fundamental role in case selection. This article presents a clinical case of central pancreatectomy to contribute to the understanding of its role as a therapeutic option in the treatment of low-grade pancreatic tumors and its impact on pancreatic tissue preservation.

## Case presentation

**Figure 1:**
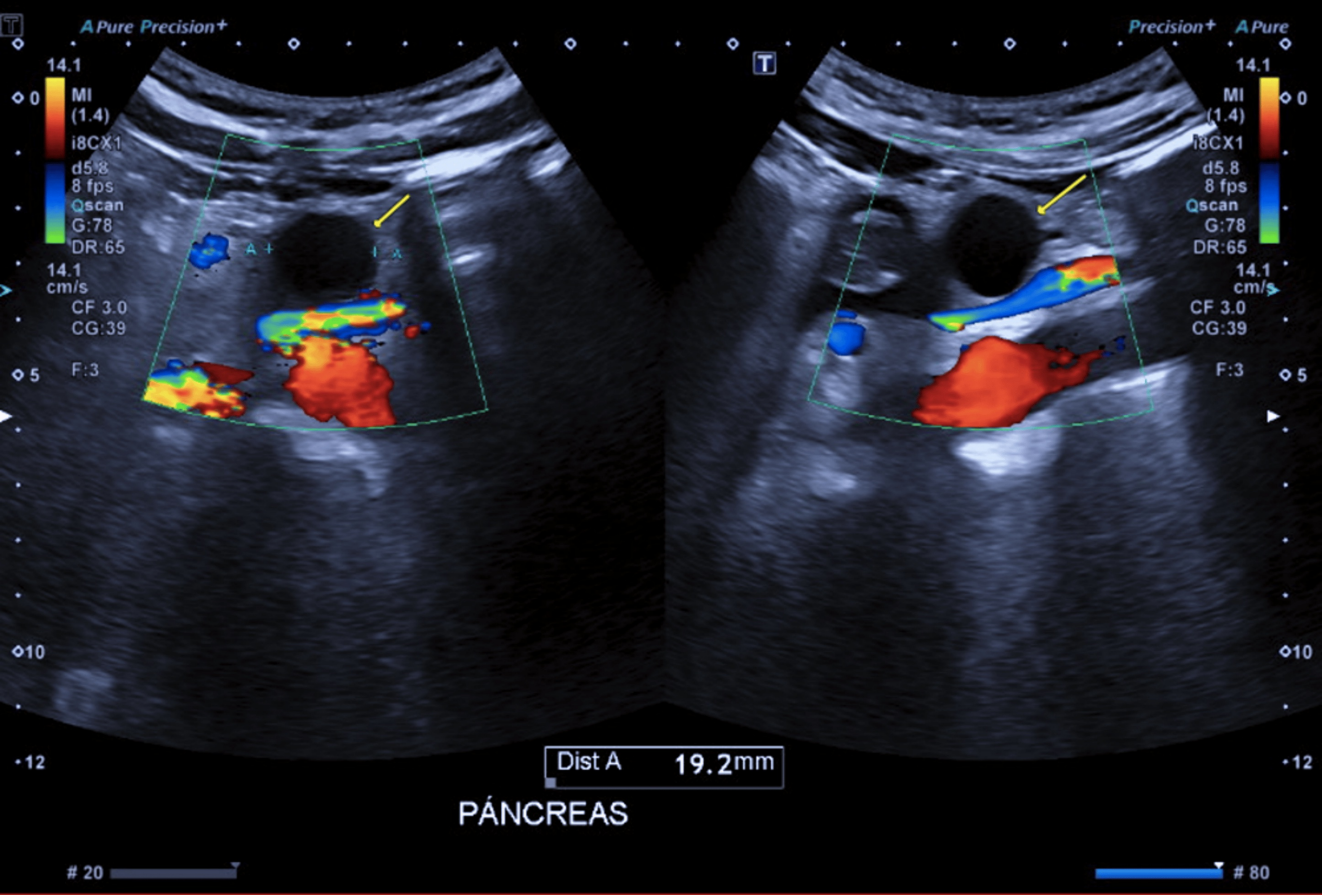
Abdominal ultrasound In the body of the pancreas, a cystic lesion with a nonaggressive appearance is identified, measuring 22 mm in its largest diameter. It does not cause retrograde dilation of the main pancreatic duct. Further evaluation with magnetic resonance imaging is recommended

A 45-year-old female patient with a history of conversion from sleeve gastrectomy to Roux-en-Y gastric bypass three years ago due to gastroesophageal reflux disease (GERD) presented with a pancreatic cystic lesion found incidentally on abdominal ultrasound screening (Figure 1).

**Figure 2:**
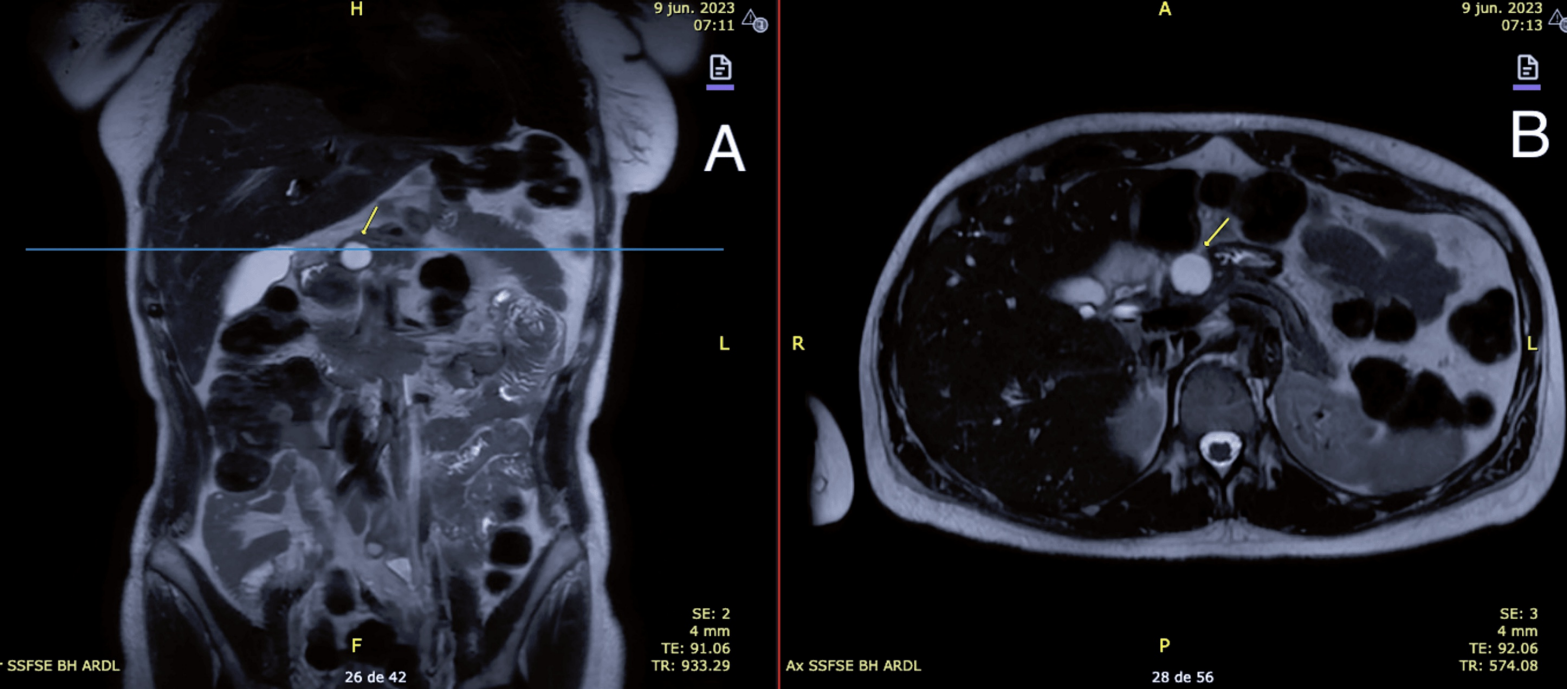
Magnetic resonance imaging (MRI): (A) coronal plane and (B) axial plane Cystic lesion in the pancreatic neck, without aggressive features, measuring 20 mm in its maximum diameter, with no clear communication with the main pancreatic duct. No other focal lesions are identified. The main pancreatic duct is of normal caliber

Further investigation with magnetic resonance imaging (MRI) revealed a 20 mm cystic lesion in the neck of the pancreas without features of malignancy (Figure 2A, 2B).

**Figure 3:**
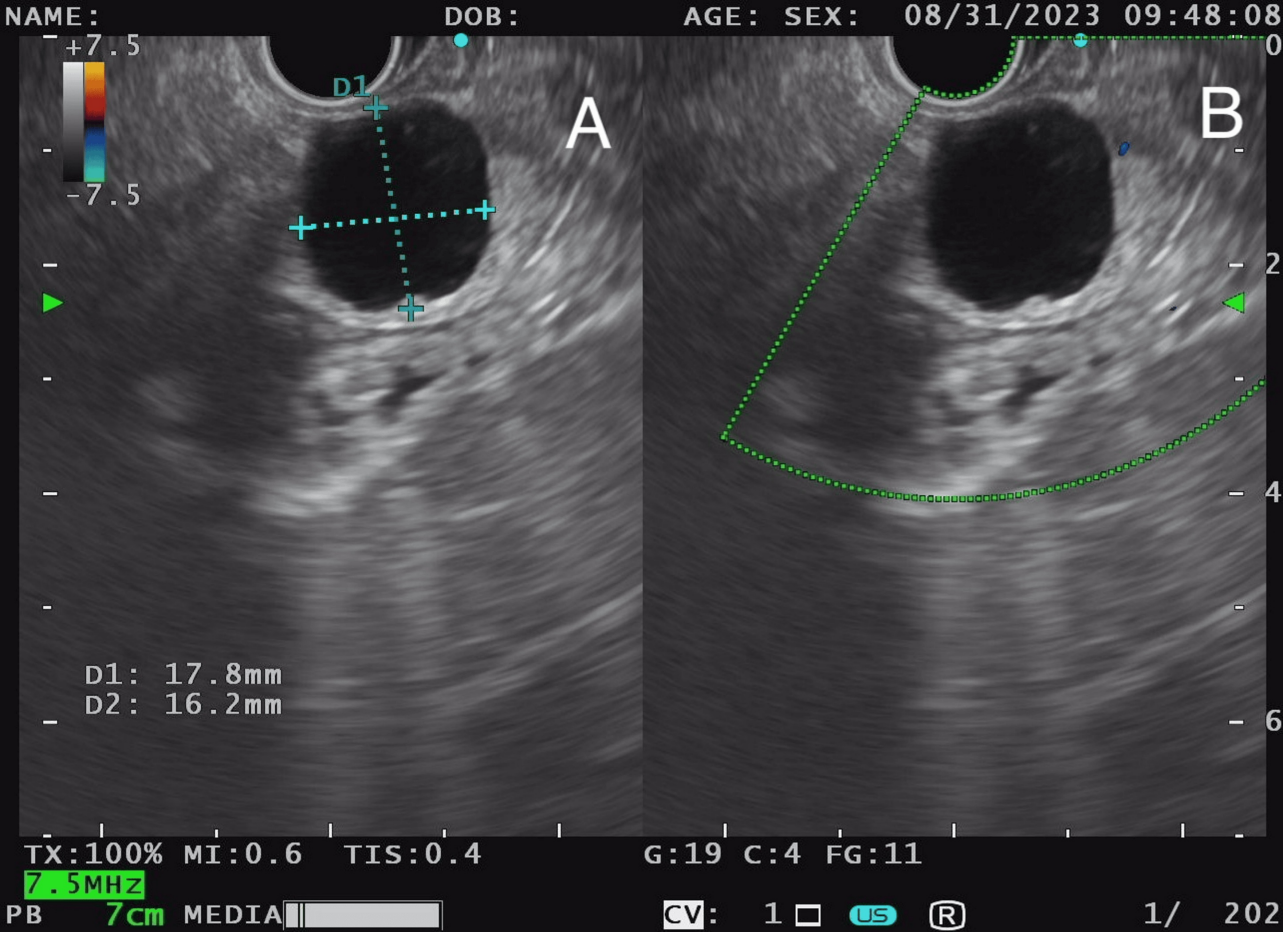
Endosonography: (A) the measurement of the pancreatic lesion and (B) the use of a 7.5 MHz transducer (A and B) Homogeneous pancreatic parenchyma with regular glandular margins. A well-defined 22 mm cyst is identified in the neck extending toward the body of the pancreas, with thin walls, no thickening or growth into the lumen, regular margins, and anechoic content

**Figure 4:**
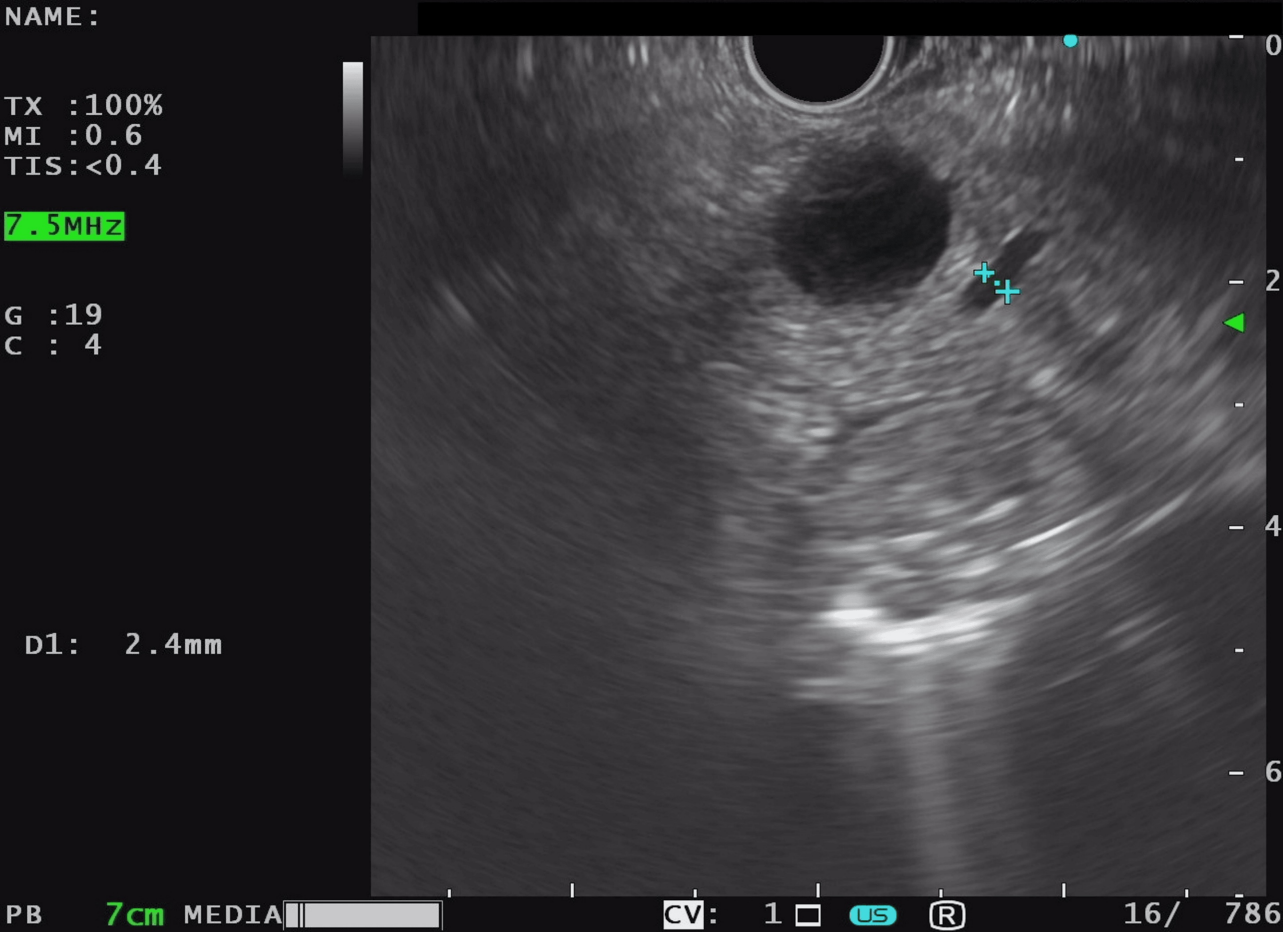
Endosonography The Wirsung duct is thin throughout its course, adjacent to the cystic lesion, which it surrounds, without any visible communication with the described lesion

Endosonography (Figure 3A, Figure 3B, and Figure 4) showed no findings suggestive of aggressiveness.

Pancreatic fine-needle aspiration (FNA) was performed (Table 1), with citrine-colored fluid aspirated and carcinoembryonic antigen (CEA) < 1.8 ng/mL. The biochemical analysis of the fluid showed amylase of 144 U/L; glucose of 102 mg/dL; immunohistochemistry, chromogranin A diffusely positive in neoplastic cells; synaptophysin, diffusely positive in neoplastic cells; and Ki-67, proliferation index estimated at <1%, consistent with well-differentiated grade 1 neuroendocrine tumor (G1 NET).

**Table 1:** Fluid analysis of the pancreatic lesion

Tests	Results	Reference range
Carcinoembryonic antigen (CEA)	<1.8 ng/mL	<5 in nonsmokers and <6.5 in smokers
Amylase	144 U/L	30-110 U/L
Glucose	102 mg/dL	Without a report of normal values
Chromogranin A	Diffusely positive in neoplastic cells	-
Synaptophysin	Diffusely positive in neoplastic cells	-
Ki-67	Proliferation index estimated at <1%	-

Preoperative laboratory tests were performed, showing serum chromogranin A within the normal reference range (Table 2).

**Table 2:** Preoperative laboratory tests

Tests	Results	Reference range
Hemoglobin	10.4 g/dL	11.7-16 g/dL
White cell count	4.5 x 10^3^ uL	4.5-11 x 10^3^ uL
Platelet count	261 x 10^3^ uL	140-400 x 10^3^ uL
Serum creatinine	0.57 mg/dL	0-5-0.9 mg/dL
C-reactive protein (CRP)	1.01 mg/dL	0.1-0.5 mg/dL
Lipase	50 U/L	13-60 U/L
International normalized ratio (INR)	1.0	-
Serum chromogranin A	89 ng/mL	108 ng/mL

**Figure 5:**
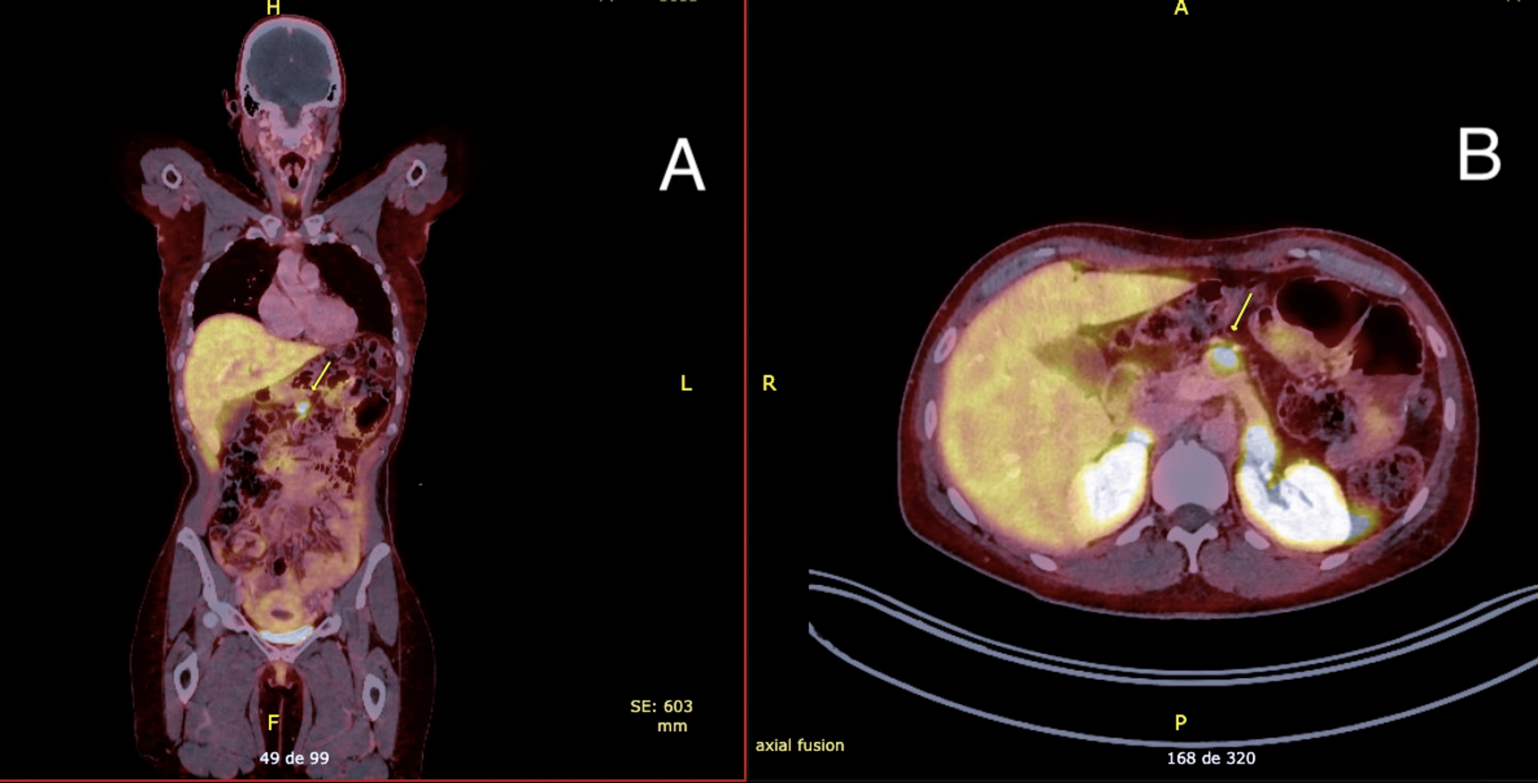
PET-CT with octreotide: (A) coronal plane and (B) axial plane Arrows: hyperuptake in the pancreas (maximum SUV: 7.5) with no other lesions, consistent with neuroendocrine neoplasia SUV: standardized uptake value

PET-CT with octreotide (Figure 5) showed uptake in the pancreas with no other lesions.

Given the suspicion of a neuroendocrine pancreatic neoplasm, the case was discussed in a clinical committee, and an open central pancreatectomy was decided.

**Figure 6:**
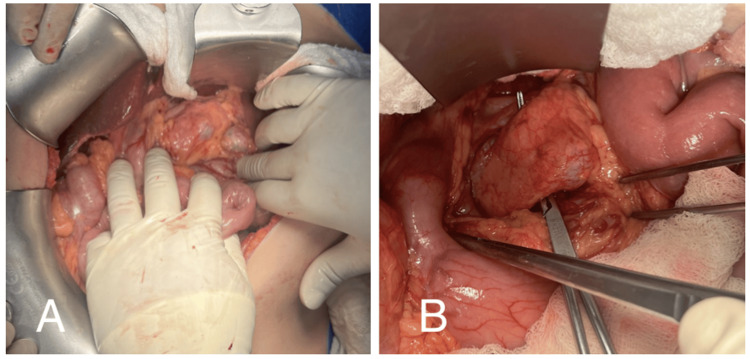
(A) Well-defined, partially exophytic cystic neoplastic lesion located in the neck of the pancreas. (B) Creation of a retro-pancreatic tunnel Complete exposure of the pancreatic neck, achieving both distal and proximal margins

The surgery was performed through a midline laparotomy, with the opening and section of the gastrocolic ligament, providing access to the lesser sac and full exposure of the pancreas. Macroscopically, a soft pancreas with a well-defined, partially exophytic cystic lesion in the neck, approximately 20 mm in its largest diameter, was identified, involving almost the entire thickness of the pancreatic parenchyma (Figure 6A).

The lymph node dissection of group 8 was performed, along with the dissection of the common hepatic artery and splenic artery. The dissection of the pancreatic groove to the left of the mesenteric vessels allowed the creation of a retro-pancreatic tunnel without complications. To the left, the splenic vein was identified in its usual position. The tunnel was completed using blunt dissection and an esophageal retractor to encircle the pancreatic body, achieving wide proximal and distal margins (Figure 6B).

A macroscopic view revealed a well-defined, partially exophytic cystic neoplasm located in the neck of the pancreas. The pancreatic neck was fully exposed, ensuring wide distal and proximal margins.

**Figure 7:**
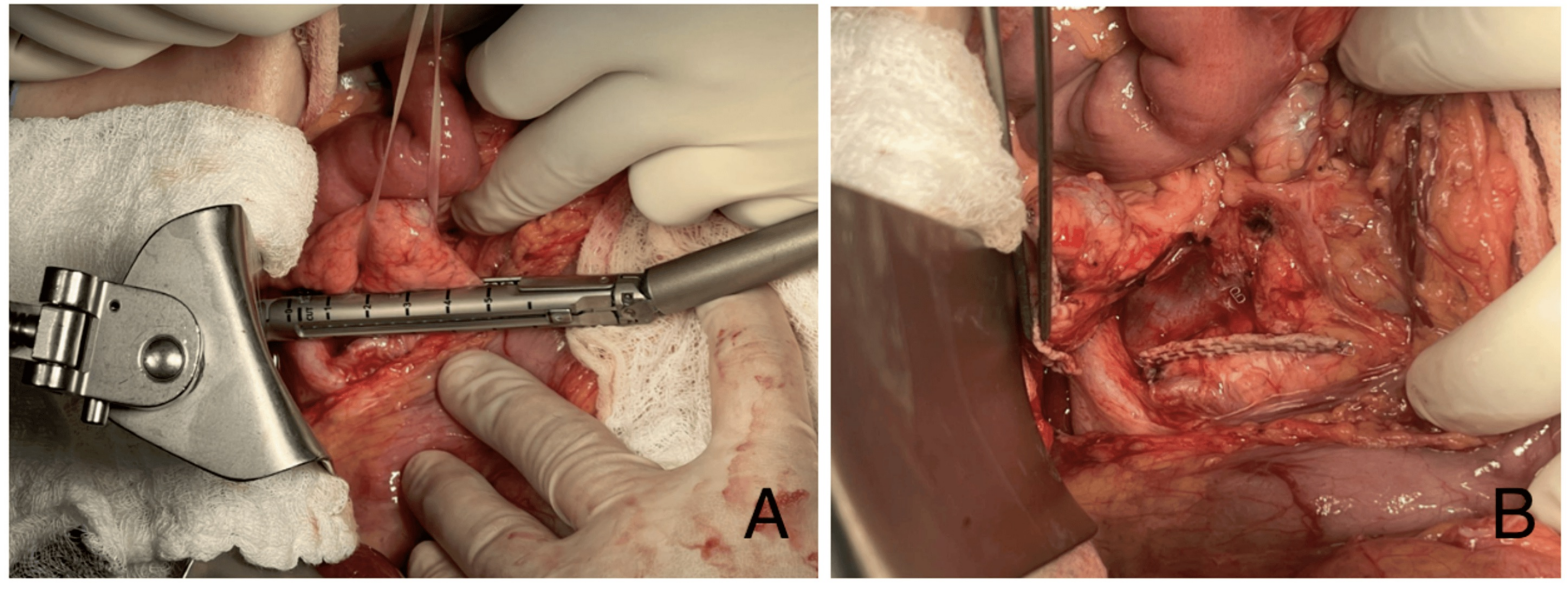
(A) Proximal transection of the pancreas using an Endo GIA 60 mm purple cartridge. (B) View of the pancreas after proximal transection

The transection of the pancreatic neck was performed using an Endo GIA (Covidien, Dublin, Ireland) 60 mm purple cartridge, and distal pancreas resection was completed with monopolar energy (Figure 7A, 7B).

**Figure 8:**
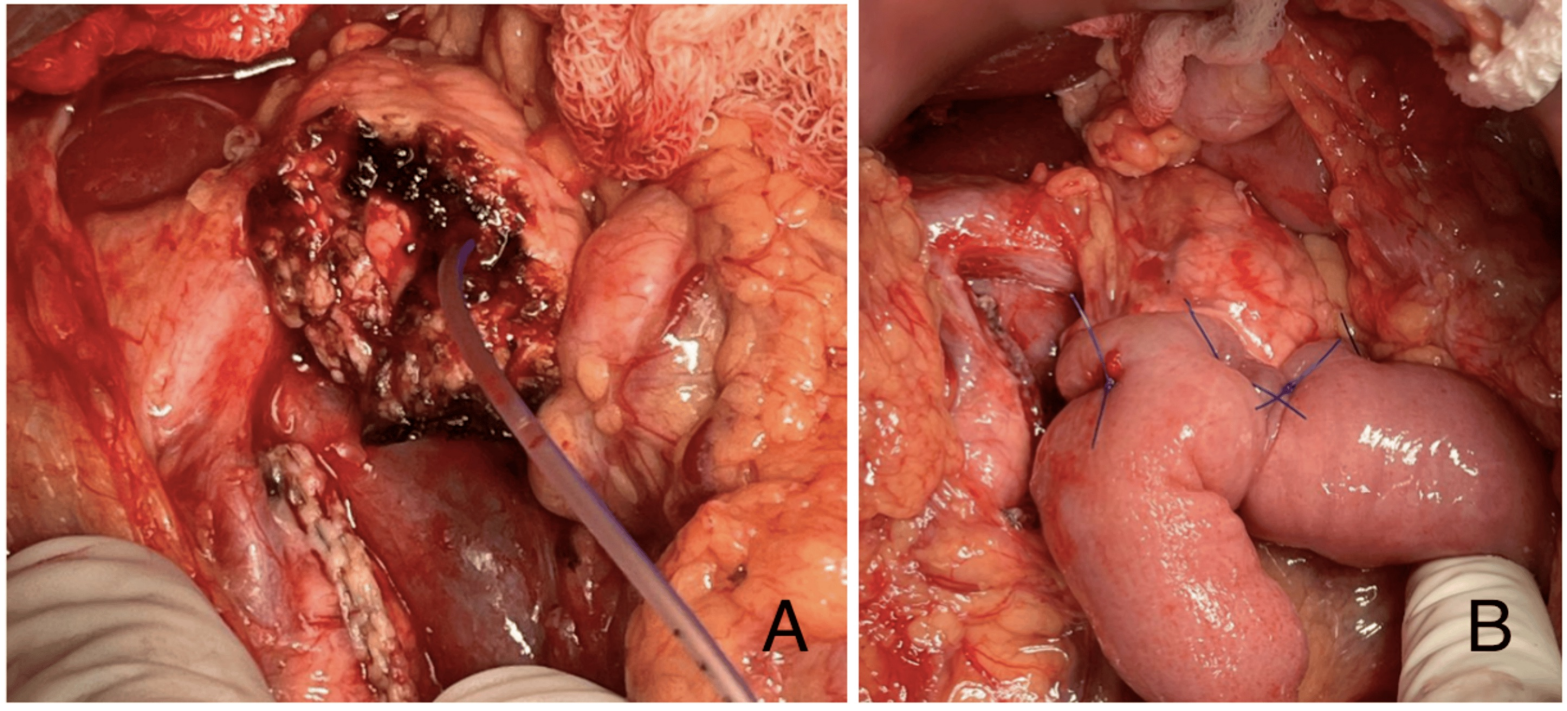
(A) Distal pancreas resection with monopolar energy. (B) Creation of a pancreatojejunostomy

The Wirsung duct was identified with a diameter of approximately 2-3 mm (Figure 8A). The jejunum was transected 20 cm distal to the previous entero-entero anastomosis of the gastric bypass. A transmesocolic loop was brought up, and a Blumgart pancreatojejunostomy was created with 10 separate duct-to-mucosa Prolene 5-0 stitches (Figure 8B). A side-to-side mechanical entero-entero anastomosis was performed.

Two Blake drains were placed in the pancreatic bed, with distal ends adjacent to the pancreatic stump and exteriorized through the right flank. On postoperative day 4, both drains had minimal output (drain I, 10 cc; drain II, 59 cc) with amylase levels of 5878 and 59 mg/dL, respectively (Table 3).

**Table 3:** Postoperative amylase levels in drainage fluid

Tests	Results	Reference range
Amylase drain I	5878 mg/dL	<3 times the serum amylase value
Amylase drain I	59 mg/dL	<3 times the serum amylase value
Serum amylase	82 mg/dL	28-100 mg/dL

Drain II was removed, and the patient was discharged on postoperative day 5 due to favorable clinical progress. She was evaluated eight days post-discharge; drain I output was 3-5 cc/day, leading to its removal.

Biopsy confirmed a well-differentiated 2.1 cm G1 neuroendocrine tumor. Surgical margins were negative, with no vascular, lymphatic, or perineural invasion (pT2N0). At the five-month follow-up, the patient was asymptomatic, with a control abdominal PET-CT showing no abnormalities.

## Discussion

Traditional approaches, such as distal pancreatectomy and pancreatoduodenectomy, involve more extensive resections, which may result in a higher risk of postoperative complications, including diabetes and malabsorption [1]. Authors such as Iacono et al. (2013) support the idea that central pancreatectomy may be superior to distal pancreatectomy in certain contexts, particularly for patients with benign or low-grade tumors. Central pancreatectomy offers greater pancreatic tissue preservation and a lower rate of severe complications compared to distal pancreatectomy, making it the preferred surgical option in selected cases [2]. The choice of surgical technique should consider not only the type of tumor but also the patient's clinical characteristics and the surgical context. Multidisciplinary clinical evaluation is essential for decision-making, where surgical planning plays a fundamental role.

In terms of pancreatic function preservation, a recent study by Lee et al. (2020) directly compared central pancreatectomy, distal pancreatectomy, and duodenopancreatectomy. Central pancreatectomy demonstrated significant advantages in terms of pancreatic functional preservation, with favorable long-term outcomes not only for pancreatic function but also in preserving pancreatic mass, which ultimately translates into a lower incidence of postoperative diabetes and an improved quality of life for patients [3].

Regarding the effectiveness and safety of central pancreatectomy, a recent systematic review and meta-analysis established its feasibility for both open and minimally invasive techniques. Although minimally invasive techniques offer additional benefits in terms of reducing surgical trauma, recovery time, and hospital stay, central pancreatectomy remains effective in open surgery [4,5]. The adoption of minimally invasive techniques developed in recent years represents a current challenge, with laparoscopic approaches being a safe technique and an important advancement in surgical practice [6].

## Conclusions

Despite the numerous advantages of central pancreatectomy, the technique requires a high level of skill and experience from the surgeon, which may limit its application in centers with less experience in pancreatic surgery. In our case, the surgical team had extensive experience in pancreatic surgery, enabling central pancreatectomy to be considered as a therapeutic option, with thorough preoperative evaluation and planning. We believe that continuous training in minimally invasive techniques and the establishment of standardized protocols play a fundamental role in improving surgical outcomes in these cases. The reviewed studies support central pancreatectomy as a valid therapeutic option and, in certain cases, a preferred choice, particularly with the current advancements in minimally invasive techniques.

## References

[1] Santangelo M, Esposito A, Tammaro V (2016). What indication, morbidity and mortality for central pancreatectomy in oncological surgery? A systematic review. Int J Surg.

[2] Iacono C, Verlato G, Ruzzenente A (2013). Systematic review of central pancreatectomy and meta-analysis of central versus distal pancreatectomy. Br J Surg.

[3] Lee DH, Han Y, Byun Y, Kim H, Kwon W, Jang JY (2020). Central pancreatectomy versus distal pancreatectomy and pancreaticoduodenectomy for benign and low-grade malignant neoplasms: a retrospective and propensity score-matched study with long-term functional outcomes and pancreas volumetry. Ann Surg Oncol.

[4] Xia N, Li J, Wang Q (2024). Safety and effectiveness of minimally invasive central pancreatectomy versus open central pancreatectomy: a systematic review and meta-analysis. Surg Endosc.

[5] Hajibandeh S, Hajibandeh S, Mowbray NG, Mortimer M, Shingler G, Kambal A, Al-Sarireh B (2024). Minimally invasive versus open central pancreatectomy: a systematic review and meta-analysis. Ann Hepatobiliary Pancreat Surg.

[6] Bi S, Liu Y, Dai W (2023). Effectiveness and safety of central pancreatectomy in benign or low-grade malignant pancreatic body lesions: a systematic review and meta-analysis. Int J Surg.

